# Reply

**Published:** 1873-01

**Authors:** 


### REPLY.

"Observer," a contributor to a western health journal, accuses us of an inconsistency of expression in an article in the last number of this journal, entitled "Medicine and Doctors." He interprets our meaning to be that, a "tyro in the use of medicine, is just as likely to be successful in the treatment of disease as the thoroughly educated M. D." This announcement startled us somewhat, and we hastened to read our article in print, to see whether it really did differ so much from what we remembered to have written. We fail, to see by what manner of argument "Observer" can so misconstrue the article referred to.

The idea we meant to have conveyed, and which we think is sufficiently plain, is, that medicines receive more credit for the cure of disease than they are entitled to. In proof of this assertion, we referred to the reputed success of practitioners of the several systems or "schools" of medicine, all differing in theory and practice, yet all claiming to cure their patients. And, as their patrons get well, whether they take sugar pellets or the more potent agencies of the regular, and as *both* systems cannot be right, even the eyes of a tyro are sufficient to discern that the patients recover "in spite of the treatment," rather than from any beneficial results obtained therefrom.

*Therefore*, we urged upon our readers to select none but educated physicians, and so escape the liklihood of being compelled to take medicines when none are indicated or required; believing that every liberally educated and observing physician is less prone to the administration of medicines, than those medical gentlemen, who are usually graduated "M. D's" by virtue of our defective system of medical education, rather than by any eminent intelligence of their own.

